# Regulation of the acetylcholine/α7nAChR anti-inflammatory pathway in COVID-19 patients

**DOI:** 10.1038/s41598-021-91417-7

**Published:** 2021-06-04

**Authors:** Alice Courties, Jeremy Boussier, Jérôme Hadjadj, Nader Yatim, Laura Barnabei, Hélène Péré, David Veyer, Solen Kernéis, Nicolas Carlier, Frédéric Pène, Frédéric Rieux-Laucat, Bruno Charbit, Vincent Bondet, Darragh Duffy, Francis Berenbaum, Benjamin Terrier, Jérémie Sellam

**Affiliations:** 1 Sorbonne Université, INSERM UMR 938, Centre de Recherche Saint-Antoine, Hôpital Saint-Antoine, AP-HP, Paris, France; 2 Rheumatology Department, AP-HP Saint-Antoine Hospital, 184, rue du Faubourg Saint-Antoine, 75012 Paris, France; 3 Sorbonne Université, Paris, France; 4 Translational Immunology Lab, Department of Immunology, Institut Pasteur, 75015 Paris, France; 5 Université de Paris, Imagine Institute Laboratory of Immunogenetics of Pediatric Autoimmune Diseases, INSERM UMR 1163, 75015 Paris, France; 6 Department of Internal Medicine, National Reference Center for Rare Systemic Autoimmune Diseases, AP-HP, APHP-CUP, Hôpital Cochin, 75014 Paris, France; 7 Université de Paris, INSERM, U970, PARCC, 75015 Paris, France; 8 Laboratoire de Virologie, Service de Microbiologie, Hôpital Européen Georges Pompidou, Assistance Publique-Hôpitaux de Paris, 75015 Paris, France; 9 Unité de Génomique Fonctionnelle des Tumeurs Solides, Centre de Recherche des Cordeliers, INSERM, Université Paris, 75005 Paris, France; 10 Equipe Mobile d’Infectiologie, Hôpital Cochin, AP-HP, APHP-CUP, 75014 Paris, France; 11 Université de Paris, INSERM, IAME, 75006 Paris, France; 12 Institut Pasteur, Epidemiology and Modelling of Antibiotic Evasion (EMAE), 75015 Paris, France; 13 Department of Pulmonology, Hôpital Cochin, AP-HP, APHP-CUP, 75014 Paris, France; 14 Université de Paris, Institut Cochin, INSERM U1016, CNRS UMR 8104, Service de Médecine Intensive-Réanimation, Hôpital Cochin, AP-HP-CUP, 75014 Paris, France; 15 Institut Pasteur, Cytometry and Biomarkers UTechS, CRT, 75015 Paris, France

**Keywords:** Acute inflammation, Viral infection

## Abstract

The cholinergic system has been proposed as a potential regulator of COVID-19-induced hypercytokinemia. We investigated whole-blood expression of cholinergic system members and correlated it with COVID-19 severity. Patients with confirmed SARS-CoV-2 infection and healthy aged-matched controls were included in this non-interventional study. A whole blood sample was drawn between 9–11 days after symptoms onset, and peripheral leukocyte phenotyping, cytokines measurement, RNA expression and plasma viral load were determined. Additionally, whole-blood expression of native alpha-7 nicotinic subunit and its negative dominant duplicate (CHRFAM7A), choline acetyltransferase and acetylcholine esterase (AchE) were determined. Thirty-seven patients with COVID-19 (10 moderate, 11 severe and 16 with critical disease) and 14 controls were included. Expression of CHRFAM7A was significantly lower in critical COVID-19 patients compared to controls. COVID-19 patients not expressing CHRFAM7A had higher levels of CRP, more extended pulmonary lesions and displayed more pronounced lymphopenia. COVID-19 patients without CHRFAM7A expression also showed increased TNF pathway expression in whole blood. AchE was also expressed in 30 COVID-19 patients and in all controls. COVID-19-induced hypercytokinemia is associated with decreased expression of the pro-inflammatory dominant negative duplicate CHRFAM7A. Expression of this duplicate might be considered before targeting the cholinergic system in COVID-19 with nicotine.

## Introduction

Coronavirus disease 19 (COVID-19) is characterized by clinical heterogeneity, with severity ranging from asymptomatic patients to critical and life-threatening disease^1^. Severe and critical COVID-19 are characterized by an acute respiratory distress syndrome (ARDS) driven by an exacerbated inflammatory response that can lead to multi-organ failure and death^2^. This hypercytokinemia, initially named “cytokine storm”, is associated with high levels of circulating tumor necrosis factor (TNF) and interleukin-6 (IL-6), profound peripheral blood lymphopenia and chemoattraction of mononuclear cells within the lungs^3–5^. Overall, this imbalanced inflammatory response is responsible for tissue damage and disease severity.

A wide variety of regulatory mechanisms have been implicated in the pathophysiology of COVID-19 hyperinflammation. Among them, the role of the cholinergic system is evoked as a counter-regulatory mechanism. It was first proposed from the observation of a small proportion of smokers among patients with symptomatic COVID-19 suggesting a potential protective role of nicotine in COVID-19^6^. Since early 2000s, animal and experimental models have shown that the cholinergic system and the parasympathetic vagus nerve reduced cytokine production^7,8^. This phenomenon involves binding of acetylcholine (Ach) to acetylcholine receptor type 7 (α7nAChR), one of the nicotinic receptors expressed on macrophages^9^. Activation of the NF-κB pathway and production of pro-inflammatory cytokines (especially TNF, IL-1β, IL-6 and IL-18) are inhibited by the binding of Ach (or nicotine) to α7nAChR^8–10^. This cholinergic anti-inflammatory pathway (CAP) thus represents a neuro-immune target in chronic inflammatory diseases^11–15^. Some authors have suggested that activation of cholinergic system through α7nAChR activation, using electrical vagus nerve stimulation or exogenous α7nAChR ligand exposure (such as nicotine or pharmacological compounds) could represent innovative therapeutic strategies to limit COVID-19-induced hypercytokinemia^6,16–19^. However, to date, while cholinergic system manipulation is being evaluated in COVID-19 patients, data on its expression and regulation during COVID-19 are scarce and restricted to infectivity of airway epithelial cells, as nicotine could promote cellular entrance of SARS-CoV-2 through α7nAChR mechanisms^20^. Before targeting the cholinergic system in COVID-19 patients, it is critical to determine the expression of the components of the cholinergic system in COVID-19 patients.

Hence, we aimed to investigate whole-blood expression of cholinergic system members and correlated it with COVID-19 severity and cytokine expression using an integrated immune analysis on a cohort of patients with COVID-19 healthy aged-matched controls^21^.

## Results

### Characteristics of the population

Thirty-seven patients infected by SARS-CoV-2 and 14 healthy controls (HC) were included (Table 1). Patients with critical COVID-19 were older and had more comorbidities such as hypertension, diabetes and/or cardiovascular diseases in comparison with patients with moderate COVID-19 patients or controls^21^. Additionally, CRP levels and viral load increased with the severity of the disease while lymphocyte counts decreased. Only one COVID-19 patient was an active smoker. Blood sampling was performed after a median duration of 10 days (interquartile range 9 to 11 days), at the usual time of hypercytokinemia.

**Table 1:** Characteristics of the whole population.

	Healthy controls *n* = 14	Moderate COVID *n* = 10	Severe COVID *n* = 11	Critical COVID *n* = 16
Age	50.4 ± 13.2	55.5 ± 12.5	55.63 ± 10.5	62.4 ± 9.9
**Sex**				
Women, n (%)	4 (28.5)	2 (20)	1 (9)	4 (25)
Men, n (%)	10 (71.5)	8 (80)	10 (91)	12 (75)
Hypertensive, n (%)	0 (0)	2 (20)	3 (27)	8 (50)
Diabetes	0 (0)	0 (0)	1 (9)	5 (31)
**Smoking status**				
Never, n (%)	14 (100)	7 (70)	10 (91)	12 (75)
Has stopped, n (%)	0	2 (20)	1 (9)	4 (25)
Current, n (%)	0	1 (10)	0 (0)	0 (0)
Cardiovascular history	0 (0)	0 (0)	0 (0)	3 (18.8)
**COVID-19 symptoms**	NA			
Fever		10 (100)	11 (100)	16 (100)
Cough		9 (90)	10 (90.9)	15 (93.7)
Dyspnea		10 (100)	11 (100)	16 (100)
Fatigue		9 (90)	11 (100)	16 (100)
Myalgia		9 (90)	8 (72.7)	6 (37.5)
Diarrhea		4 (90)	6 (54.5)	1 (6.2)
**Extent of CT lung lesions**				
< 10%, *n* (%)		4 (40)	0	0
10–25%, *n* (%)		4 (40)	3 (27.3)	3 (18.75)
25–50%, *n* (%)		1 (10)	8 (72.7)	6 (37.5)
50–75%, *n* (%)		1 (10)	0	4 (25)
Not available		0	0	3 (18.75)
Lymphocytes/mm^3^, mean ± SD	NA	1198 ± 407	941.8 ± 320.4	771.3 ± 312.3
CRP levels (mg/L), mean ± SD	1.2 ± 1.6	38.2 ± 32.5	170.1 ± 82.2	234.4 ± 123.0
**Plasma viral load, mean ± SD**				
N Gene (cp/mL plasma)	NA	325.4 ± 731.8	299.2 ± 239.1	2476 ± 6558

*NA* not applicable or not available, *SD* standard deviation.

### Whole-blood expression of the Ach/α7nAChR pathway in COVID-19

**Figure 1:**
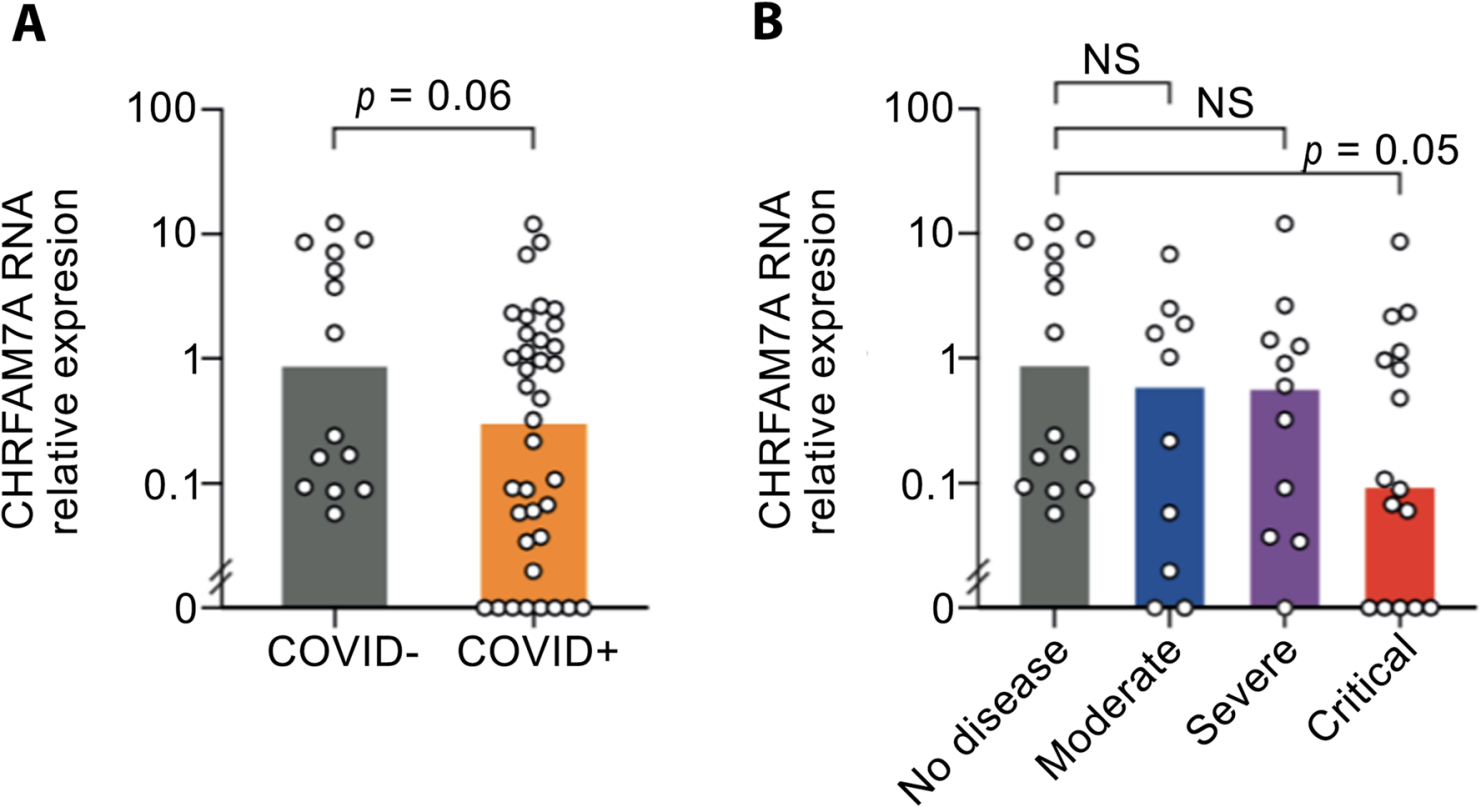
Expression of CHRFAM7A on whole blood controls and COVID-19 patients. RNA expression of the dominant negative duplicate CHRFAM7A in whole blood depending on COVID infection (**A**, *n* = 14 controls, 37 infected) and according to COVID-19 severity (**B**), determined by quantitative polymerase chain reaction (qPCR). *p < 0.05; *TNF* tumor necrosis factor.

Among molecular actors of the cholinergic system, whole-blood mRNA expression of the native *Chrna7* subunit was undetectable in patients as well as in healthy controls. In contrast, 21 (57%) COVID-19 patients and 10 (71%) healthy controls expressed the human dominant negative duplicate *CHRFAM7A*. COVID-19 patients tended to have a decreased expression of *CHRFAM7A* compared to healthy controls (1.35 ± 2.5 versus 3.45 ± 4.2, *p* = 0.06) (Fig. 1A). Stratifying patients according to COVID-19 severity, *CHRFAM7A* expression was significantly lower in critical COVID-19 patients compared to HC (3.45 ± 4.2 versus 1.05 ± 2.2, *p* = 0.045) (Fig. 1B). Smoking status did not influence the expression of cholinergic genes, notably in former smokers. Similarly to *Chrna7*, ChAT expression was not detectable in patients and HC. In contrast, AChE mRNA was detected in 30 (81%) COVID-19 patients and in all (100%) controls (*p* = 0.07).

### Comparison of COVID-19 patients according to CHRFAM7A expression

To further study the relationship between the whole-blood expression of CHRFAM7A and disease severity, we compared COVID-19 patients expressing (*n* = 21) and those not expressing CHRFAM7A (*n* = 16). Despite a younger age, COVID-19 patients not expressing CHRFAM7A subunit showed features related to more severe disease, including higher level of C-reactive protein (*p* = 0.04) and more pronounced lymphopenia (*p* = 0.05 for total lymphocyte, *p* = 0.03 for % live lymphocytes) (Table 2). The proportion of critical patients within the group of patients that did not express CHRFAM7A was higher than in those that expressed CHRFAM7A (50% versus 38.1%) but the difference did not reach statistical significance. However, we observed a trend of a higher proportion of patients with extension pulmonary lesions (> 25%) in the CT-Scan in patients with no expression of CHRFAM7A compared to those expressing CHRFAM7A (73.3% versus 44.4% respectively, *p* = 0.09).

**Table 2:** Comparison of COVID-19 patients according mRNA expression of CHRFAM7A in whole blood.

	CHRFAM7A+ *n* = 21	CHRFAM7A− *n* = 16	*p*-value*
Age, mean ± SD	61.8 ± 12.7	54.4 ± 7.5	0.0062
**Sex**			
Women, n (%)	5 (28.5%)	2 (12.5%)	NS
Men, n (%)	15 (71.4%)	14 (87.5%)	
**Severity**			
Mild/moderate, n (%)	6 (28.6%)	4 (25%)	NS
Severe, n (%)	7 (33.3%)	4 (25%)	
Critical, n (%)	8 (38.1%)	8 (50%)	
**TDM extension lesion**			
< 25%, n (%)	10 (55.6%)	4 (26.7%)	0.09
> 25%, n (%)	8 (44.4%)	11 (73.3%)	
CRP levels (mg/L), mean ± SD	119.2 ± 81	223.9 ± 142.2	0.04
AchE (2^−∆∆CT^), mean ± SD	3.18 ± 5.5	8.47 ± 21.9	NS
**Cells, mean ± SD**			
Leucocytes/mm^3^	7019 ± 3137	7728 ± 5228	NS
Lymphocytes/mm^3^	1023 ± 339.5	814.4 ± 390.7	0.05
Lymphocytes, %	18.1 ± 1 9.4	13.0 ± 6.5	0.03
CD3/mm^3^	694.0 ± 239.8	540.8 ± 346.1	0.04
CD3%	12.6 ± 6.9	9.1 ± 6.4	0.02
B cells, %	2.36 ± 1.4	2.27 ± 0.98	NS
CD19/mm^3^	157.8 ± 110	155.1 ± 124.3	NS
Th2/Th1 ratio	0.74 ± 0.57	0.94 ± 0.49	NS
NK/mm^3^	170.3 ± 124.6	101.4 ± 51.6	NS
pDC/mm^3^	1.7 ± 1.5	1.48 ± 0.94	NS
Monocytes/mm^3^	536.5 ± 346.4	413.1 ± 283.6	NS
**ELISA cytokines, mean ± SD**			
IL6 (pg/mL)	37.5 ± 28.3	62.9 ± 50.0	NS
TNF (pg/mL)	3.6 ± 1.8	4.4 ± 1.7	NS
IL10 (pg/mL)	7.4 ± 1.4	7.9 ± 2.3	NS
IL17A (pg/mL)	21.1 ± 21.7	21.9 ± 16.9	NS
IL1β (pg/mL)	1.85 ± 2.35	1.1 ± 2.6	NS
IFNγ (fg/mL)	1093 ± 1983	1456 ± 2213	NS
IFNα2c (fg/mL)	7545 ± 12,648	2962 ± 2827	NS
**Plasma viral load, mean ± SD**			
N gene (cp/mL plasma)	484.5 ± 634.3	2135 ± 6409	NS

*N* number, *NS* not significant, *pg/mL* picogram per milliliter, *fg/mL* femtogram per milliliter, *cp/mL* copy number per milliliter, *pDC* peripheric dendritic cells, *NK* natural killer, *IL* interleukin, *IFNγ* interferon gamma, *IFNα2c* interferon alpha 2c. *p-value were calculated using Mann–Whitney test, and are specified if ≤ 0.1; otherwise NS is reported.

### Correlation between CHRFAM7A and TNF or IL6 in COVID-19 patients and controls

**Figure 2:**
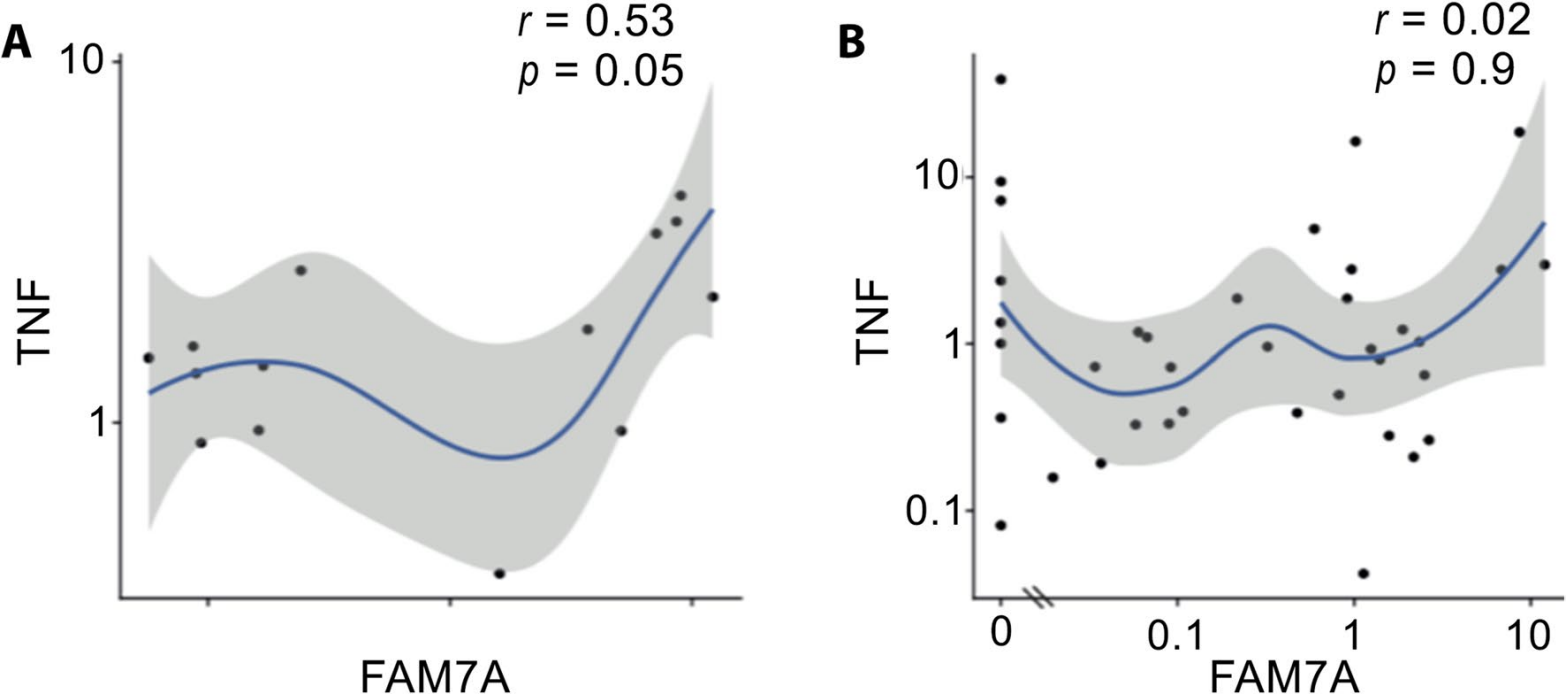
Correlation of CHRFAM7A and TNF mRNA whole blood expression in controls and COVID-19 patients. Spearman’s correlation of qPCR expression between TNF and CHRFAM7A in controls (**A**, *n* = 14) and in COVID19 patients (**B**, *n* = 37).

TNF and IL6 were previously shown to be among the master cytokines involved in COVID-19 related hyperinflammation. Therefore, we analyzed the relationship between expressed members of the cholinergic system and TNF and IL6 mRNA and protein expressions. *CHRFAM7A* expression correlated with mRNA TNF expression (*r* = 0.53, *p* = 0.054) in healthy controls, while in COVID-19 patients such a *CHRFAM7A*/*TNF* correlation was not observed (*r* = 0.02, *p* = 0.9) (Fig. 2A,B). However, there was no correlation between mRNA *CHRFAM7A* and TNF protein expression assessed by digital ELISA in COVID-19 as well as in HC subjects. IL6 mRNA and protein expression did not correlate with *CHRFAM7A* mRNA expression in both COVID-19 and HC (*data not shown*). We observed that TNF and IL6 mRNA levels did not correlate with TNF and IL6 protein levels respectively, suggesting that cytokines are coming from infected tissues rather than blood cells which may explain why CHRFAM7A did not correlate with ELISA cytokines levels.

### Molecular signature associated with CHRFAM7A expression in COVID-19 patients

**Figure 3:**
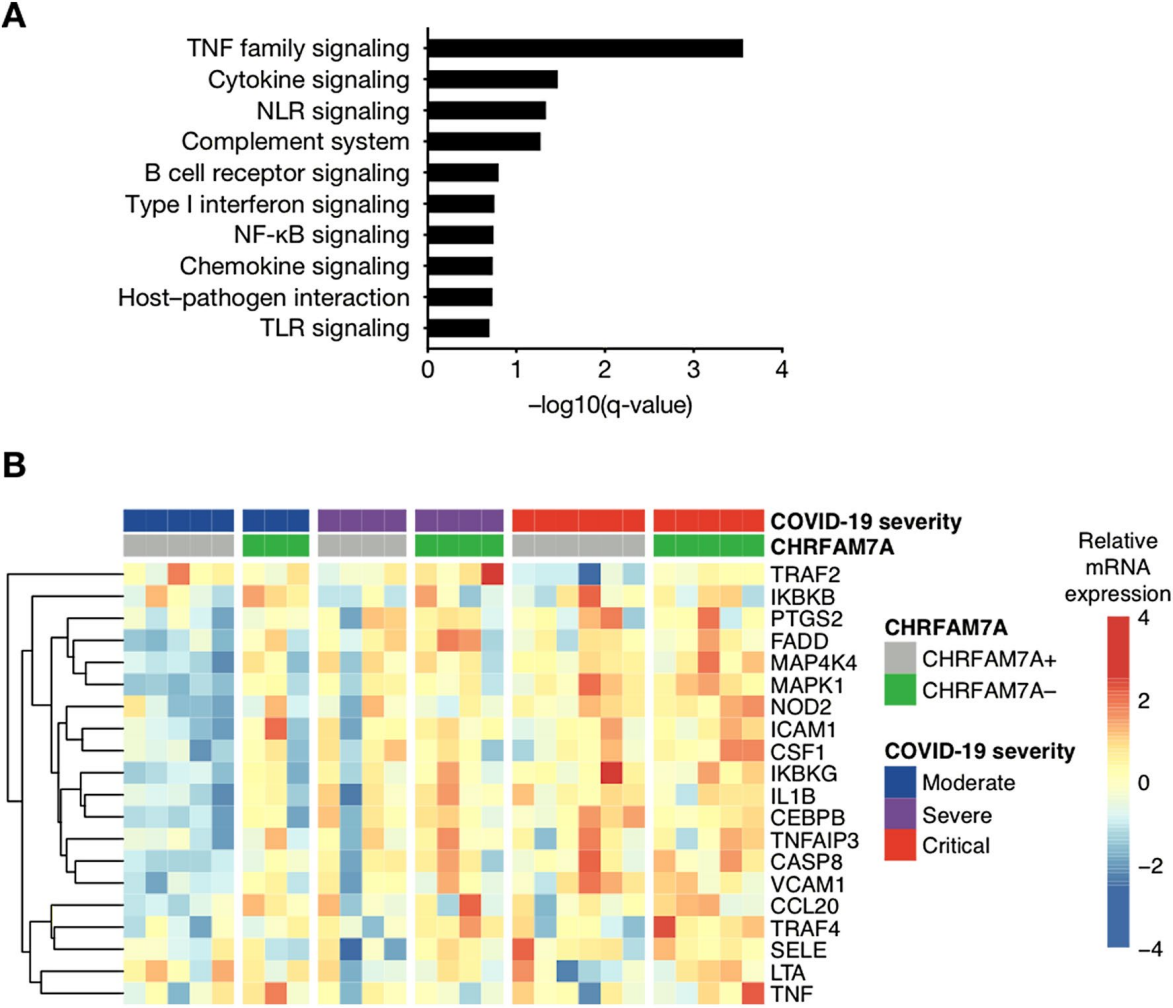
Pathway enrichment analysis between COVID-19 patients according to the expression of CHRFAM7A, the negative dominant duplicate of the alpha 7 nicotinic subunit. RNA was extracted from patient whole blood and RNA counts of 574 genes were determined by means of direct probe hybridization, using the Nanostring nCounter Human Immunology_v2 kit. (**A**) Gene set enrichment analysis was performed after ranking genes according to their differential expression in CHRFAM7A+ versus CHRFAM7A− patients. Shown are pathways significantly enriched (false discovery rate < 0.2). (**B**) Heatmap showing expression of 20 most differentially expressed TNF-related genes in patients with mild-to-moderate (*n* = 8), severe (*n* = 8), and critical (*n* = 11) SARS-CoV-2 infection according to their positive or negative expression of CHRFAM7A. Up-regulated genes are shown in red, and down-regulated genes in blue.

We analyzed whole-blood RNA levels of 574 genes using the Nanostring technology to determine gene expression differences between COVID-19 patients expressing or not CHRFAM7A. Gene set enrichment analysis identified several significantly enriched pathways (Fig. 3A, GSEA enrichment score with false discovery rate < 0.2), with increased gene expression in CHRFAM7A− compared to CHRFAM7A+ patients. The most enriched pathway was TNF family signaling. Heatmap analysis revealed that the difference between the groups was mostly mediated by the moderate group, and to a lesser extent by the severe group (Fig. 3B).

## Discussion

Since observational data suggested that the cholinergic system could be involved in COVID-19-related hypercytokinemia and hyperinflammation, we investigated whole-blood expression of the main markers of the Ach/α7nAChR pathway in COVID-19.

We found that CHRFAM7A expression was decreased in COVID-19 patients, whereas most controls expressed the dominant negative CHRFAM7A duplicate. Also, this decrease was related to disease severity of the infection. Absence of CHRFAM7A expression in COVID-19 patients was associated with some increased inflammatory biological markers and COVID-19 severity (lymphopenia, elevated CRP, elevated plasma viral load) with extension of the pulmonary lesions and with enhanced expression of genes of the TNF signaling pathway. These results suggest that during COVID19-related hypercytokinemia, decrease of the pro-inflammatory duplicate CHRFAM7A could be proposed as a compensatory process to counteract the inappropriate inflammatory response. This may also explain why TNF levels positively correlated with CHRFAM7A in controls but not in COVID-19 patients. The loss of this correlation could be a consequence of the dysregulation of the control of cholinergic system during inflammation. As shown by the heatmap analysis, the association between the absence of CHFRAM7A expression and the increase in TNF signaling pathway was observed for patients with moderate and severe COVID-19 patients but not for those with critical disease. If we hypothesize that the decrease or absence of CHRFAM7A is a compensatory mechanism to make the produced ACh more efficient, it is possible that this mechanism is exceeded in overly severe patients. Of course, we cannot make a causal link or confirm the compensatory aspect of expression because only one sample per patient at one time point was available. Longitudinal analysis will be required to fully understand modulation of the cholinergic system during COVID-19. Another explanation could be that patient with intrinsic decreased or absence of CHRFAM7A expression have higher risk to develop a symptomatic and severe COVID-19.

Additionally, we found that AchE was expressed by almost all controls and patients. The enzymatic activity of AchE might be important to evaluate before manipulating the cholinergic system in COVID-19 patients. We did not find any significant expression of the native Chrna7 subunit, which was not surprising since the expression of the native Chrna7 subunit is mostly present in the neuronal system and resident cells and much less in the non-neuronal system, especially in the circulating cells^22,23^. ChAT expression was not detectable either in whole-blood RNA, which could be due to the method of detection since we analyzed whole-blood expression and not specific expression after cell sorting.

Although these results are observational and cannot testify for a causal relationship, they provide elements for further discussion about cholinergic system manipulation in COVID-19. While clinical trials investigating the efficacy of vagus nerve stimulation or nicotine administration in COVID-19 for cholinergic anti-inflammatory pathway activation are ongoing, the impact of the expression of the dominant negative duplicate CHRFAM7A has never been considered. Yet, this duplicate has been implicated for explaining the previous failures of pharmacological α7 nicotinic receptor agonists in neurocognitive diseases, while murine studies were clearly encouraging^24^. Knowing that trials are underway to evaluate the usefulness of nicotine on the risk of developing a SARS-CoV-2 infection and severe COVID-19, it seems critical to address the influence of the dominant negative duplicate CHRFAM7A expression on the nicotine response.

Finally, this work has some limitations. Unfortunately, we do not have longitudinal samples to demonstrate whether regulation is related to the disease state and we did not performed heart rate variability measurement using a long-electrocardiogram to characterize the vagal tone in these patients^25^. However, comparison of COVID-19 patients accurately phenotyped with controls may provide some indications about biological cholinergic disturbances induced by the disease. We found a difference in age between subjects expressing and those not expressing CHRFAM7A, which could have influenced the inflammatory markers, age being a determining factor of severity. However, this difference was in the opposite direction since patients not expressing CHRFAM7A were younger while they exhibited more features of disease severity. Second, it would be interesting to confirm these data in tissues damaged by COVID-19 such as the lung. However, blood leukocytes are crucial in the anti-inflammatory signal induced by the cholinergic anti-inflammatory pathway and blood seems appropriate to investigate hypercytokinemia which is a systemic phenomenon. It would also be interesting to study in vitro the monocytic and lymphocytic regulation of the expression of the CHRFAM7A duplicate by inflammatory cytokines.

In conclusion, COVID-19-related hypercytokinemia shows some correlations with regulation of the Ach/α7nAChR pathway characterized by a decreased expression of the pro-inflammatory dominant negative duplicate CHRFAM7A. The expression of this duplicate should be considered in clinical trials evaluating therapeutic strategies manipulating the cholinergic system in COVID-19 such as nicotine.

## Methods

### Study population

This study is derived from another study that evaluated immune response in 50 COVID-19 patients with various disease severity^21^. Briefly, this non-interventional study was conducted between March 19, 2020 and April 3, 2020 in Cochin Hospital (Paris, France) to explore molecular signature associated with COVID-19 severity. Adult patients with COVID-19 according to WHO interim guidance, and positive SARS-CoV-2 RT-PCR testing on a respiratory sample (nasopharyngeal swab or invasive respiratory sample) were included. Inpatients with preexisting unstable chronic disorders and with bacterial co-infection were excluded. Healthy controls (HC) were asymptomatic adults, matched with cases on age (± 5 years), with a negative nasal SARS-CoV-2 RT-PCR testing at time of inclusion. Biological collection and informed consent were approved by the Direction de la Recherche Clinique et Innovation (DRCI) and the French Ministry of Research (N°2019-3677). The study conforms to the principles outlined in the Declaration of Helsinki and received approval by the appropriate Institutional Review Board (Cochin Port Royal Hospital, Paris, France; number AAA-2020-08018). Demographic, clinical, biological, CT-scan data were extracted from the electronical medical files. The severity of COVID-19 was classified at the time of admission based on the adaptation of the Sixth Revised Trial Version of the Novel Coronavirus Pneumonia Diagnosis and Treatment Guidance between moderate, severe or critical. Written informed consent was obtained for all participants. A whole blood sample was performed between 8 and 12 days from the beginning of the symptoms as it is the crucial period for development of respiratory failure and cytokine storm.

### Whole blood analyses

An extensive analysis was performed on the whole blood as previously published to determine peripheral blood leukocytes phenotyping using mass cytometry, gene expression profile using Nanostring, cytokines measurement by digital ELISA and Luminex and quantification of viral load by digital PCR^21^.

### RNA and quantitative PCR analysis of the cholinergic system

Reverse transcription was performed using 500 ng of total RNA with an Omniscript RT kit (Qiagen). Levels of mRNA for Chrna7, CHRFAM7A, ChAT and AChE as well as of IL6 and TNF were quantified by quantitative RT-PCR using a Light-Cycler LC480 (Roche Diagnostics) after 40 cycles of amplification. A number > 38 cycles was considered as negative. Levels of mRNA were normalized to those of GADPH. This relative quantitative value is equal to 2^−∆CT^ where ∆CT is equal to Ct target minus Ct of GAPDH. The sequence of the primers have been already published^15^.

### Statistical analysis

Quantitative data are expressed as the mean ± SD or median [interquartile] and qualitative as n (%). Mann–Whitney test was used for comparisons of quantitative data and Chi-squared test of independence was used to compare qualitative data between groups. *P*-values ≤ 0.05 were considered significant. Spearman correlation was used to assess correlation. Statistical analyses were performed using GraphPad Prism 8 and R (CRAN) v. 3.4.3.
